# Retraction Note: KiVa anti-bullying program: preventing and reducing bullying behavior among students– a scoping review

**DOI:** 10.1186/s12889-025-22342-x

**Published:** 2025-03-19

**Authors:** Rohman Hikmat, Suryani Suryani, Iyus Yosep, Rohani Jeharsae

**Affiliations:** 1 Master Program of Nursing, Faculty of Nursing, Universitas Padjadjaran, Sumedang, Jawa Barat Indonesia; 2 Department of Mental Health, Faculty of Nursing, Universitas Padjadjaran, Jawa Barat, Sumedang, Indonesia; 3 Faculty of Nursing, Prince of Songkhla University, Pattani Campus, Rusamilae, Thailand

**Retraction Note: BMC Public Health (2024) 24:2923**

10.1186/s12889-024-20086-8

The Editors have retracted this article after concerns were raised about its representation of the KiVa anti-bullying program. For example, this article states that the KiVa program includes a system of anonymised complaints and that it incorporates self-therapy and meditation techniques, though these are not features of the program. Concerns have also been raised that the article misrepresents the content of several studies cited as part of this review. For example, reference no. 2 does not mention bullying prevalence in Estonia, Russia or Japan, as is claimed in the article, and reference no. 41 reports a study conducted with students in grades 3–5, not grades 8–9 as is claimed in the article. The Editors no longer have confidence in the findings of this review.

Suryani Suryani agrees with this retraction. The remaining authors did not respond to correspondence from the Publisher regarding this retraction.

